# Construction and validation of a risk prediction model for oral frailty in elderly patients with esophageal cancer

**DOI:** 10.3389/fonc.2025.1736063

**Published:** 2026-01-13

**Authors:** Jingwen Lv, Jianwei Li, Yu Wang, Sipeng Liu, Yunhong Du, Li Wang, Hui Wang, Yao Shi

**Affiliations:** 1 Intensive Care Unit, The First Affiliated Hospital of Naval Medical University, Shanghai, China; 2 Nursing Department, Qingdao Traditional Chinese Medicine Hospital (Qingdao Hiser Hospital Affiliated of Qingdao University), Qingdao, China; 3 School of Nursing, Hunan University of Chinese Medicine, Changsha, China; 4 Nanchang University Queen Mary School, Nanchang, China; 5 Neurology Department, The First Affiliated Hospital of Naval Medical University, Shanghai, China; 6 Cardiovascular Surgery Intensive Care Unit, The First Affiliated Hospital of Naval Medical University, Shanghai, China

**Keywords:** elderly patients, esophageal cancer, nomogram, oral frailty, risk prediction model

## Abstract

**Background:**

To explore the current status of oral frailty in elderly patients with esophageal cancer (EC), construct and verify the risk prediction model of oral frailty in elderly patients with EC, so as to provide a reference for early identification and intervention of oral frailty in this population.

**Methods:**

In this study, 390 elderly patients with EC treated in the First Affiliated Hospital of Naval Medical University from January, 2023 to June, 2024 were selected as the training group, and 165 elderly patients with EC treated in Qingdao Hiser Hospital Affiliated of Qingdao University from July, 2024 to July, 2025 were selected as the validation group. A cross-sectional study was used for data collection. Three types of assessment tools were used, including the self-made general information questionnaire, outcome variable assessment scale and candidate variable assessment scale. The patients were divided into two groups according to the occurrence of oral frailty. LASSO regression and multivariate analysis were performed using SPSS 27.0 and R 4.4.3 software to identify the independent risk factors for oral frailty. The Bootstrap method with 1000 repeated samplings was used for internal validation of the model, while external validation was conducted using data from the validation group. The performance of the model was evaluated by the area under the receiver operating characteristic curve (AUC), calibration curve, Hosmer-Lemeshow test, decision curve analysis (DCA) and other indicators, and the Nomogram was drawn to visualize the model.

**Results:**

The incidence of oral frailty in the training group and validation group was 45.90% and 43.03%, respectively. The results showed that radiotherapy history, tumor staging, physical frailty, smoking history, age, and nutritional status were independent risk factors for oral frailty in elderly patients with EC (*P<* 0.05). The area under the ROC curve of the training group and the validation group were 0.812 (95% CI: 0.771–0.853) and 0.796 (95% CI: 0.730–0.862), respectively. Hosmer-Lemeshow test results (χ^2^ = 12.382, *P* = 0.193) and (χ^2^ = 14.922, *P* = 0.093) indicated that the model had a good goodness of fit. The consistency between the actual value of the calibration curve and the predicted value was high. The DCA results suggested that the model could obtain net benefits within a large threshold probability in both internal and external validation.

**Conclusion:**

The risk of oral frailty in elderly patients with EC is high, which is related to radiotherapy history, tumor staging, physical frailty, smoking history, age, nutritional status and other factors. The oral frailty risk prediction model for elderly patients with EC constructed in this study has good predictive efficacy in internal and external validation, which can provide a reference for medical staff to identify high-risk groups early and take targeted intervention measures.

## 1 Introduction

With the accelerating global population aging, oral frailty, as a crucial phenotype of geriatric syndromes, has garnered increasing attention. Its characteristics include (1) age-related oral changes in individuals, such as reduced number of teeth, poor oral hygiene, and impaired oral function, often accompanied by decreased interest in oral health, diminished physical and psychological reserve capacity, and eating difficulties. This condition directly affects patients’ nutritional intake, verbal communication, and quality of life, and exhibits a high degree of correlation with a series of adverse health outcomes, including malnutrition, increased risk of systemic infections, and elevated mortality rates (2–6).

As one of the most prevalent malignant tumors worldwide, esophageal cancer (EC) has a relatively high incidence in the elderly population (7–9). Due to space-occupying lesions of the tumor, side effects of radiotherapy and chemotherapy, and anatomical and physiological correlations, patients often develop oral dysfunctions such as impaired oral self-cleansing ability, oral mucositis, and dysphagia (10, 11). In addition, elderly patients are frequently comorbid with multiple chronic diseases, and the interaction between their global frailty status and oral function decline renders elderly EC patients a high-risk group for oral frailty.

Although numerous studies on oral frailty and its influencing factors have been conducted domestically and internationally, there remains a significant gap in the risk assessment and management of oral frailty among elderly patients in current clinical diagnosis, treatment, and nursing practice for EC. Most existing studies are limited to single-dimensional assessment of oral function or global frailty, lacking systematic research on the synergistic mechanisms between oral frailty and global frailty. Furthermore, there is currently no standardized oral frailty risk prediction tool suitable for EC-specific clinical settings, making it difficult to achieve early identification and precise intervention of high-risk individuals.

By integrating multi-dimensional influencing factors, risk prediction models can scientifically and quantitatively predict the probability of disease or adverse outcome, and provide a reliable basis for clinical decision-making (12). In view of this, on the basis of previous studies, combined with the disease characteristics and pathogenesis of oral frailty in elderly patients with EC, this study intends to systematically screen the key predictive variables, so as to construct an accurate risk prediction model for oral frailty in elderly patients with EC and provide effective assessment methods for clinical medical staff to identify high-risk groups early. And lay a theoretical foundation for the subsequent development of targeted intervention measures.

## 2 Methods

### 2.1 Study design

A cross-sectional study design was used, involving elderly patients with EC treated in two tertiary hospitals in China (the First Affiliated Hospital of Naval Medical University and Qingdao Hiser Hospital Affiliated of Qingdao University). In this study, we strictly followed the ethical principles the Declaration of Helsinki (13), ensured that the rights of participants were fully protected. It did not pose any risk or harm to patients and was implemented after the review and approval of the hospital ethics committee (Ethics batch number: 2022HCO8LS010).

### 2.2 Sample size calculation

The formula based on sample size in epidemiological cross-sectional studies was used in this study (14), $n=\frac{Z_{1-\alpha /2}^{2}\cdot P\cdot (1-P)}{d^{2}}$. According to the pre-survey, the risk of frailty in elderly patients with EC was 33%, and d was the allowable error. In this study, the allowable error was controlled at 5%, and the two-sided test method was used under the premise of assuming the CI is 95%, so the Z_1-α/2_ in the formula was 1.96; *P* = 0.30; d = 0.05. A sample size of at least 378 in the training group was also taken into account for a sample missing rate of 10%. The sample size of the risk prediction model validation group was generally 1/4–1/2 of the sample size of the training group (15), invalid questionnaires were 10%, so the sample size of the validation group was at least 105.

### 2.3 Study subjects

Using the convenient sampling method, a total of 390 elderly patients with EC treated in the First Affiliated Hospital of Naval Medical University from January, 2023 to June, 2024 were selected as the training group, and 165 elderly patients with EC treated in Qingdao Hiser Hospital Affiliated of Qingdao University from July, 2024 to July, 2025 were selected as the validation group.

(I) Inclusion criteria: Patients with EC who met the diagnostic criteria of the Guidelines for the Diagnosis and Treatment of Esophageal Cancer (2022 edition) (16); age ≥60 years old; communication skills without obvious cognitive impairment, able to clearly express their wishes; stable condition; voluntarily participated in the study and signed the informed consent form, and voluntarily accepted the relevant investigation of the study.

(II) Exclusion criteria: Patients with serious primary diseases of liver, kidney, hematopoietic system, endocrine system and other serious organ failure; unable to eat by mouth; with organic diseases of salivary glands or Sjogren’s syndrome; severe vision and hearing impairment.

(III) Rejecting standard: Poor questionnaire completion quality (data missing rate >10%); poor compliance and distorted results due to emotion or other reasons; patients voluntarily withdraw from the study or sudden deterioration of the condition.

### 2.4 Research tools

#### 2.4.1 Self-made questionnaire

Self-designed, based on literature review and clinical expert opinion, this study screened potential influencing factors and constructed the General information questionnaire of oral frailty in elderly patients with EC, which consisted of general demographic data and disease-related information, including: Gender, age, smoking history, drinking history, marital status, social support level, education level, family per capita monthly income level, living status, body mass index, oral health status, denture, physical frailty, depression, history of surgery, radiotherapy, chemotherapy, tumor staging, metastasis, tumor location, histological type, nutritional status, serum albumin level.

#### 2.4.2 Variable assessment tools

(I) Patient-Generated Subjective Global Assessment (PG-SGA): It is a nutritional status assessment form specially designed for cancer patients, which uses a comprehensive method to assess their nutritional status (17). The instrument includes both patient self-assessment and provider assessment with a total score ranging from 0 to 35. Only the patient self-assessment scale part was used in this study, with higher scores indicating worse nutritional status, divided into A = well nourished (0 to 3 points), B = suspected or moderate malnutrition (4 to 8 points), and C = severe malnutrition (>8 points). The sensitivity of the scale was 87.6%, the specificity was 83.1%, and the Cronbach’s α coefficient was 0.712 (18);

(II) Geriatric Depression Scale-15 (GDS-15): contains 15 items (19), patients answered “yes” or “no” to each item, and “no” to items 1, 5, 7, and 11 was assigned 1 point, and “yes” to the other items was assigned 1 point. Total scores range from 0 to 15, with scores ≥5 indicating depression. The Cronbach’s α coefficient of the Chinese version was 0.793 (20);

(III) Social Support Rating Scale (SSRS): This scale had 10 items and 3 dimensions, namely objective support, social support utilization and subjective support (21). The total score ranges from 12 to 66 points, the score of objective support dimension was 4–16, the score of subjective support dimension was 5–38, and the score of support utilization dimension was 3–12. The higher the score, the more support the individual received. According to the score, it can be divided into three levels: the low level is 12–22 points, the middle level is 23–44 points, and the high level is 45–66 points. The scale has good reliability and validity, and the Cronbach’s α coefficient was 0.91 (21).

(IV) Oral Health Assessment Tool (OHAT): according to brief oral health checklist by Chalmers et al. (22) have been revised, the Chinese version developed and validated by Wang et al. (23), was used in this study. An eight-item questionnaire was used to assess various aspects of oral health, including lips, tongue, gingival tissue, saliva, natural teeth, dentures, oral cleanliness, and the presence of toothache. Each item was assessed in terms of current status and scored as follows: 0 for normal, 1 for change, and 2 for abnormal. Total scores range from 0 to 16, with<3 indicating good oral health status and ≥3 indicating poor oral health status. A higher total score reflects poorer oral health. The Cronbach’s α coefficient was 0.71 (23).

(V) The Frailty Screening Scale (FRAIL) was developed by geriatricians from the International Society on Nutrition, Health and Ageing (24), the Chinese version was adapted by Wei Y et al. (25). It consists of five items: fatigue, reduced resistance/endurance, reduced mobility, presence of more than five comorbidities, and weight loss. Each item is scored with 1 point, and the total score ranges from 0 to 5. A score of 0 indicates health status, a score of 1 to 2 indicates pre-frailty, and a score of 3 or higher indicates frailty. The Cronbach’s α coefficient of the original scale was 0.826 (25).

#### 2.4.3 Oral frailty assessment tools

Oral Frailty Index-8 (OFI-8): developed by Tanaka et al. (26). In this study, we used the Chinese version developed by Chen et al. (27). The five-dimension and eight-item scale includes eating hard food more difficult than six months ago, sometimes choking on tea or soup, using dentures, having a dry mouth, going out less than six months ago, being able to chew hard food, brushing teeth at least twice a day, and visiting the dentist at least once a year. Total scores range from 0 to 11, with 0 to 3 indicating no oral frailty and ≥4 indicating oral frailty. Chen et al. (27) adjusted the scale to adapt to the use in China and showed good reliability and validity, with Cronbach’s α coefficient of 0.949.

### 2.5 Statistical methods

Excel 2016 software was used for data entry, SPSS 27.0 software and R4.4.3 software were used for statistical analysis. (I) Data cleaning: after data collection, double entry and reverse check were used to input the collected research data into Excel software, and the Vlookup function and the data duplication function in Excel software were used to double check the data. If there were abnormal values, the original medical records were checked again. The cases with missing values >20% were deleted, and the rest were imputed by multiple imputation, the number of imputation was 5. The data set was selected according to the reliability analysis results. (II) Statistical description: the measurement data characteristics satisfying normality were described by mean ± standard deviation ( $\bar{\text{x}}\text{=s}$), and the t test was used for comparison between groups. The measurement data that did not meet the normality were expressed by median and interquartile range ( M,I*QR*), and the Wilcoxon rank sum test was used for comparison between groups. The characteristics of the count data were described by frequency and percentage, and the chi-square test or Fisher’s exact probability method was used for comparison between groups. (III) Model fitting: LASSO regression was used for preliminary screening of predictors. Variables with non-zero regression coefficients were subjected to multivariate analysis to identify independent variables with statistically significant differences. The Variance inflation factor (VIF) was applied to examine for multicollinearity among independent variables. Finally, the selected predictors were used to construct an oral frailty risk prediction model for elderly patients with EC, with the significance level set at α=0.05. (IV) Model evaluation: the Bootstrap repeated sampling method was used for internal validation of the model, and the data of the validation group were collected for external validation. The receiver operating characteristic (ROC) Curve was drawn and the area under the receiver operating characteristic curve (AUC) was calculated to evaluate the discrimination of the model. The calibration degree of the model was evaluated by Hosmer-Lemeshow test, calibration curve and Brier score. The Clinical practicability of the model was evaluated according to the decision curve analysis (DCA). The model was visualized in the form of a nomogram.

## 3 Results

### 3.1 Current status of oral frailty in elderly patients with EC

When collecting the data of the training group, a total of 400 questionnaires were sent out, 394 questionnaires were returned, and 390 of them were valid, with an effective recovery rate of 97.50%. There were 179 cases in the oral frailty group and 211 cases in the non-oral frailty group, and the actual incidence of oral frailty was 45.90%.

**Figure 1:**
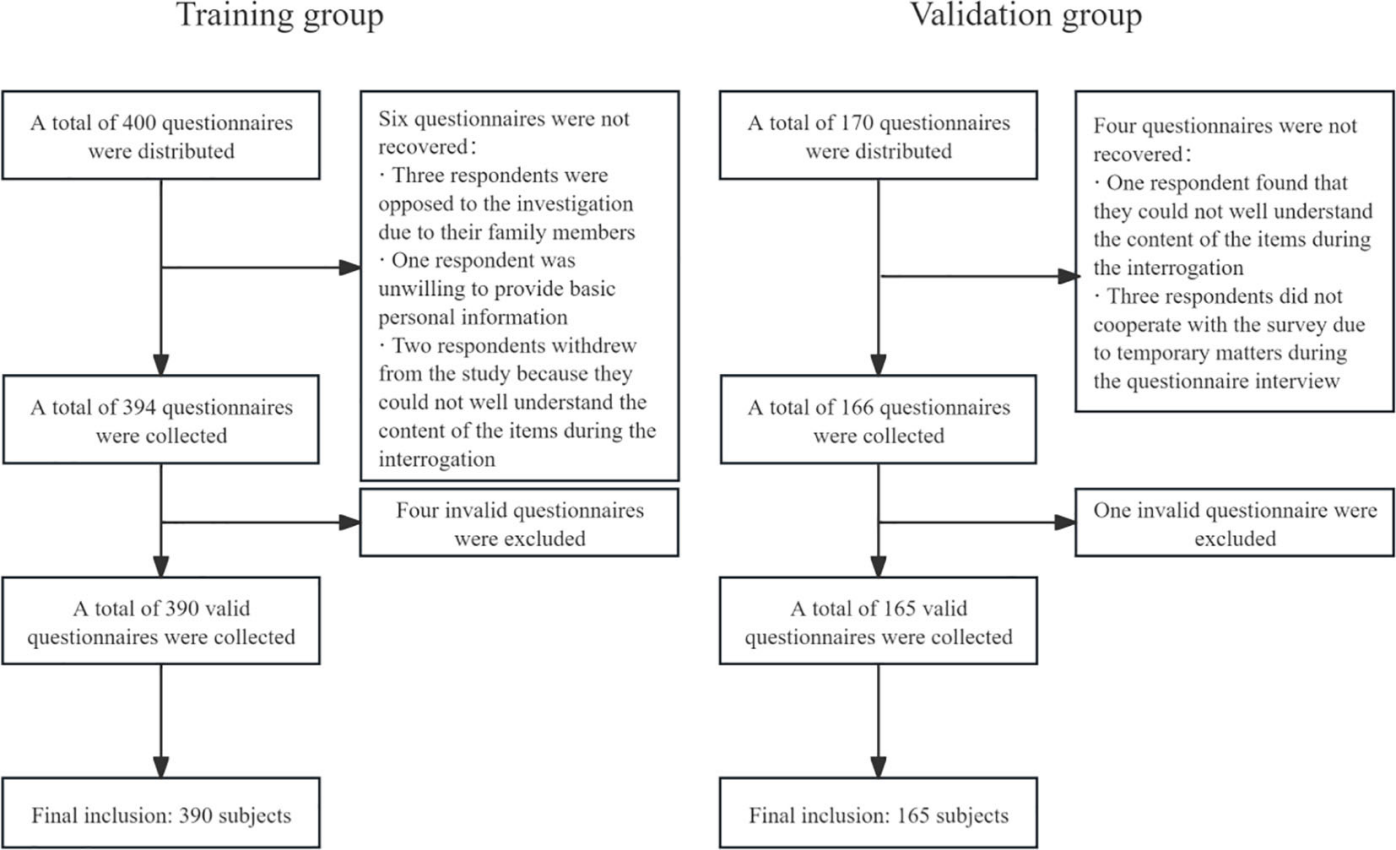
Flowchart of the inclusion process for the training group and the validation group study subjects.

When collecting the data of the validation group, a total of 170 questionnaires were distributed, 166 were returned, and 165 were valid, with an effective recovery rate of 97.06%. There were 71 cases in the oral frailty group and 94 cases in the non-oral frailty group, and the actual incidence of oral frailty was 43.03%. See **Figure 1** for details.

### 3.2 General data of elderly patients with EC

A total of 390 subjects aged 60–84 years were included in the training group, and 165 subjects aged 60–87 years were included in the validation group. There was no significant difference in general demographic data and disease characteristics between the two groups (*P >*0.05), and the baseline data were basically consistent. Details are provided in **Table 1**.

**Table 1:** Distribution of baseline characteristics in the training and validation groups [n(%)/M(*P*_25_,*P*_75_)].

Independent variable	Level	Training group (390)	Validation group (165)	Value of statistics	*P* value
Gender				0.374	0.541
	man	276 (70.8)	121 (73.3)		
	woman	114 (29.2)	44 (26.7)		
Age				0.698	0.952
	60–64 years old	81 (20.8)	35 (21.2)		
	65–69 years old	127 (32.6)	49 (29.7)		
	70–74 years old	95 (24.4)	42 (25.5)		
	75–79 years old	66 (16.9)	28 (17.0)		
	≥80 years old	21 (5.4)	11 (6.7)		
BMI				0.288	0.866
	≥24	74 (19.0)	29 (17.6)		
	18.5≤BMI<24	246 (63.1)	108 (65.5)		
	<18.5	70 (17.9)	28 (17.0)		
Nutritional status				0.880	0.644
	Good nutrition	122 (31.3)	56 (33.9)		
	Mild to moderate malnutrition	154 (39.5)	67 (40.6)		
	Severe malnutrition	114 (29.2)	42 (25.5)		
History of surgery				0.372	0.542
	No	129 (33.1)	59 (35.8)		
	Yes	261 (66.9)	106 (64.2)		
Radiotherapy history				0.036	0.849
	No	188 (48.2)	81 (49.1)		
	Yes	202 (51.8)	84 (50.9)		
Chemotherapy history				0.239	0.625
	No	94 (24.1)	43 (26.1)		
	Yes	296 (75.9)	122 (73.9)		
Tumor staging				0.075	0.784
	Stage I–II	222 (56.9)	96 (58.2)		
	Stage III–IV	168 (43.1)	69 (41.8)		
Presence or absence of metastasis				0.324	0.569
	No	284 (72.8)	124 (75.2)		
	Yes	106 (27.2)	41 (24.8)		
Location of the tumor				0.744	0.689
	Upper segment	56 (14.4)	27 (16.4)		
	Middle section	235 (60.3)	101 (61.2)		
	Lower segment	99 (25.4)	37 (22.4)		
Histologic types				0.474	0.789
	Squamous cell carcinoma	289 (74.1)	118 (71.5)		
	Adenocarcinoma	71 (18.2)	32 (19.4)		
	Other types	30 (7.7)	15 (9.1)		
Albumin				0.096	0.756
	≥35g/L	269 (69.0)	116 (70.3)		
	<35g/L	121 (31.0)	49 (29.7)		
Oral health status				0.370	0.543
	State of health	164 (42.1)	74 (44.8)		
	Ill health	226 (57.9)	91 (55.2)		
Dentures				0.548	0.459
	No	277 (71.0)	112 (67.9)		
	Yes	113 (29.0)	53 (32.1)		
Physical frailty				0.522	0.470
	No frailty or pre-frailty	141 (36.2)	65 (39.4)		
	Physical frailty	249 (63.8)	100 (60.6)		
Presence or absence of depression				0.072	0.788
	No	253 (64.9)	109 (66.1)		
	Yes	137 (35.1)	56 (33.9)		
Smoking history				0.228	0.633
	No	226 (57.9)	92(55.8)		
	Yes	164 (42.1)	73 (44.2)		
History of alcohol consumption				1.859	0.173
	No	269 (69.0)	104 (63.0)		
	Yes	121 (31.0)	61 (37.0)		
Marital status				1.042	0.307
	Married	279 (71.5)	125 (75.8)		
	Unmarried/divorced/widowed/others	111 (28.5)	40 (24.2)		
Social support level				0.155	0.925
	High level	77 (19.7)	35 (21.2)		
	Medium level	176 (45.1)	73 (44.2)		
	Low level	137 (35.1)	57 (34.5)		
Education level				0.441	0.802
	College or above	63 (16.2)	23 (13.9)		
	Junior/Senior high School	146 (37.4)	64 (38.8)		
	Primary school and below	181 (46.4)	78 (47.3)		
Monthly income level				0.523	0.770
	>5000 yuan	61 (15.6)	22 (13.3)		
	2500–5000 yuan	178 (45.6)	76 (46.1)		
	<2500 yuan	151 (38.7)	67 (40.6)		
Status of residence				0.282	0.595
	Don’t live alone	362 (92.8)	151 (91.5)		
	Live alone	28 (7.2)	14 (8.5)		

BMI, body mass index.

### 3.3 Analysis of influencing factors of oral frailty in elderly patients with EC

#### 3.3.1 LASSO regression

**Figure 2:**
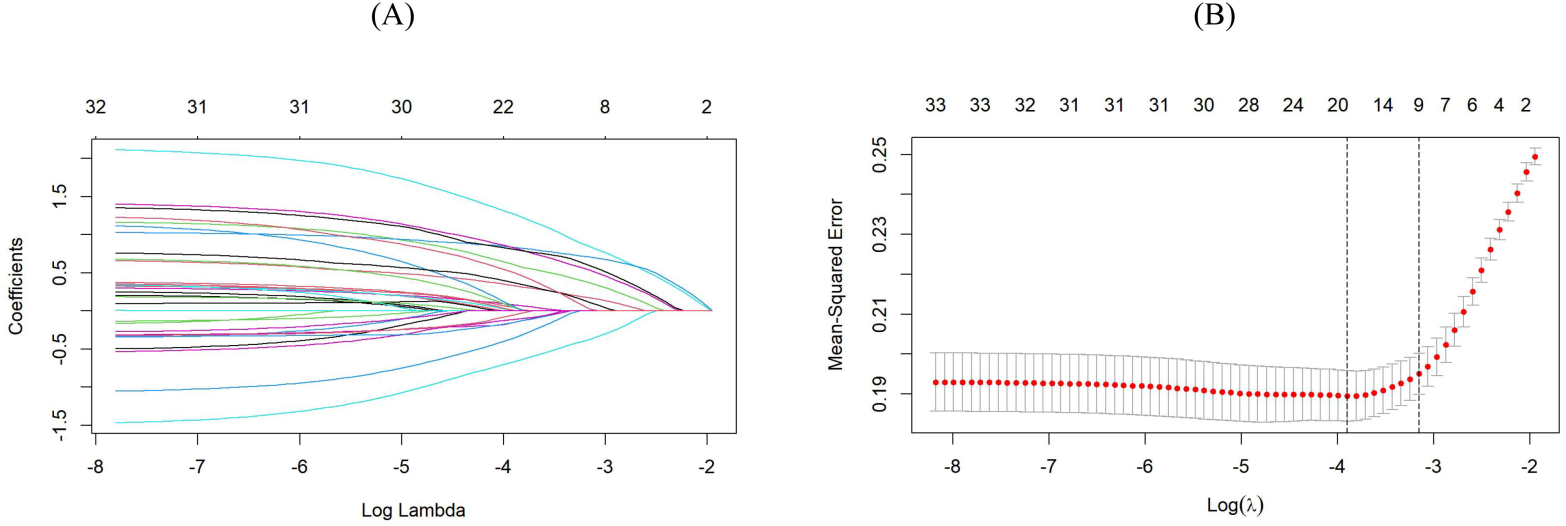
**(A)** Distribution of LASSO coefficients for each candidate variable. Each curve represents a different variable. The left ordinate denotes the coefficient values, and a coefficient of 0 indicates that the corresponding variable was not included in the model. LASSO, Least Absolute Shrinkage and Selection Operator. **(B)** Plot of Log (λ) (Logarithm of the Tuning Parameter) versus partial likelihood deviance based on LASSO Cross-Validation. At the top of the plot is the number of key feature variables corresponding to different Log (λ) values. The left dashed line represents the Log (λ) value corresponding to the minimum deviance, and the right dashed line denotes the Log (λ) value within one standard error of the minimum deviance.

In this study, LASSO regression was used for dimensionality reduction of 23 collected clinical variables to preliminarily exclude non-essential factors. To avoid overfitting, the coefficients of independent variables are gradually shrunk as the penalty parameter λ changes, until the coefficients of some variables are reduced to 0. **Figure 2A** shows the distribution of LASSO coefficients for each candidate variable during the screening process, and the final variable selection results are presented in **Figure 2B**. Based on the characteristics of the outcome variable, the predictors included in the model when λ = 0.04254021 were selected. At this point, the model comprises seven predictors, namely radiotherapy history, tumor staging, oral health status, physical frailty, smoking history, nutritional status (severe malnutrition), and age (70–74 years old, 75–79 years old, ≥80 years old).

#### 3.3.2 Multivariate analysis

With the presence or absence of oral frailty in elderly patients with EC as the dependent variable, and the seven predictors selected by LASSO regression as independent variables, multivariate analysis was performed on the training group to further screen for influencing factors. All variables were coded as dummy variables, with specific variable coding details shown in **Table 2**. The multivariate analysis indicated that radiotherapy history (OR = 2.467, 95% CI: 1.512–4.026), tumor staging III–IV (OR = 5.059, 95% CI: 3.011–8.499), physical frailty (OR = 2.937, 95% CI: 1.703–5.066), smoking history (OR = 3.291, 95% CI: 1.986–5.455), age 65–69 years old (OR = 2.477, 95% CI: 1.239–4.950), age 70–74 years old (OR = 3.111, 95% CI: 1.449–6.681), age 75–79 years old (OR = 6.282, 95% CI: 2.567–15.372), age ≥ge years old (OR = 8.324, 95% CI: 2.329–29.746), and severe malnutrition (OR = 4.391, 95% CI: 2.276–8.471) were identified as independent influencing factors for oral frailty in elderly EC patients (*P*< 0.05), with details shown in **Table 3**.

**Table 2:** Independent variable assignment table.

Independent variable	Assignment of value
Radiotherapy history	0 = no; 1 = yes
Tumor staging	1 = staging I–II; 2 = staging III–IV
Presence or absence of metastasis	0 = no; 1 = yes
Oral health status	0 = oral health; 1 = Ill health
Dentures	0 = no; 1 = yes
Physical frailty	1 = no frailty or pre-frailty; 2 = physical frailty
Smoking history	0 = no; 1 = yes
Age	1 = 60–64; 2 = 65–69; 3 = 70–74; 4 = 75–79; 5 = ≥80
BMI (Kg/m^2^)	1 = ≥24; 2 = 18.5–23.999; 3 =<18.5
Nutritional status	1 = good nutrition; 2 = mild to moderate malnutrition; 3 = severe malnutrition

**Table 3:** Logistic regression analysis of the risk of oral frailty in elderly patients with EC.

Independent variable	Grouping	β value	SE	Wald	*P* value	OR value	95% CI
Radiotherapy history		0.903	0.250	13.075	**<0.001**	2.467	1.512–4.026
Tumor staging		1.621	0.265	37.513	**<0.001**	5.059	3.011–8.499
Physical frailty		1.077	0.278	14.998	**<0.001**	2.937	1.703–5.066
Smoking history		1.191	0.258	21.354	**<0.001**	3.291	1.986–5.455
Age	60–64 years old			20.069	**<0.001**		
	65–69 years old	0.907	0.353	6.592	**0.010**	2.477	1.239–4.950
	70–74 years old	1.135	0.390	8.470	**0.004**	3.111	1.449–6.681
	75–79 years old	1.838	0.457	16.199	**<0.001**	6.282	2.567–15.372
	≥80 years old	2.119	0.650	10.636	**0.001**	8.324	2.329–29.746
Nutritional status	Good nutrition			21.342	**<0.001**		
	Mild to moderate malnutrition	0.356	0.298	1.424	0.233	1.428	0.796–2.562
	Severe malnutrition	1.480	0.335	19.473	**<0.001**	4.391	2.276–8.471
Oral health status		0.444	0.271	2.689	0.101	1.559	0.917–2.651
Constant		-6.833	0.808	71.437	**<0.001**	0.001	

SE, standard error; OR, odds ratio; 95% CI, 95% confidence interval; Values with P ≤ 0.05 were considered statistically significant (bolded in the table for the corresponding P value entries).

#### 3.3.3 Multicollinearity diagnosis

Multicollinearity among the above six variables was tested using VIF. The results showed (see **Table 4**) that the tolerance values of radiotherapy history, tumor staging, physical frailty, smoking history, nutritional status, and age were all greater than 0.1, with corresponding VIF values all less than 5. This indicates that there is no multicollinearity among the covariates, and all variables can be included in the prediction model.

**Table 4:** Collinearity diagnosis of predictors.

Variables	Collinearity tolerance	VIF
Radiotherapy history	0.996	1.004
Tumor staging	0.986	1.014
Physical frailty	0.874	1.145
Smoking history	0.998	1.002
Nutritional status	0.994	1.006
Age	0.867	1.153

VIF, Variance inflation factor.

#### 3.3.4 Establishment of prediction model for oral frailty in elderly patients with EC

The independent influencing factors that entered the multivariate analysis were sorted to construct a risk prediction model for oral frailty in elderly patients with EC: Logit (p) = (-6.833 + 0.903 × [radiotherapy]) + (1.621 × [tumor staging III–IV]) + (1.077× [physical frailty]) + (1.191 × [smoking]) + (0.907 × [age 65–69 years old]) + (1.135 × [age 70–74 years old]) + (1.838× [age 75–79 years old]) + (2.119× [age ≥80 years old]) + (1.480 × [severe malnutrition]).

#### 3.3.5 Performance evaluation of oral frailty prediction model in elderly patients with EC

**Figure 3:**
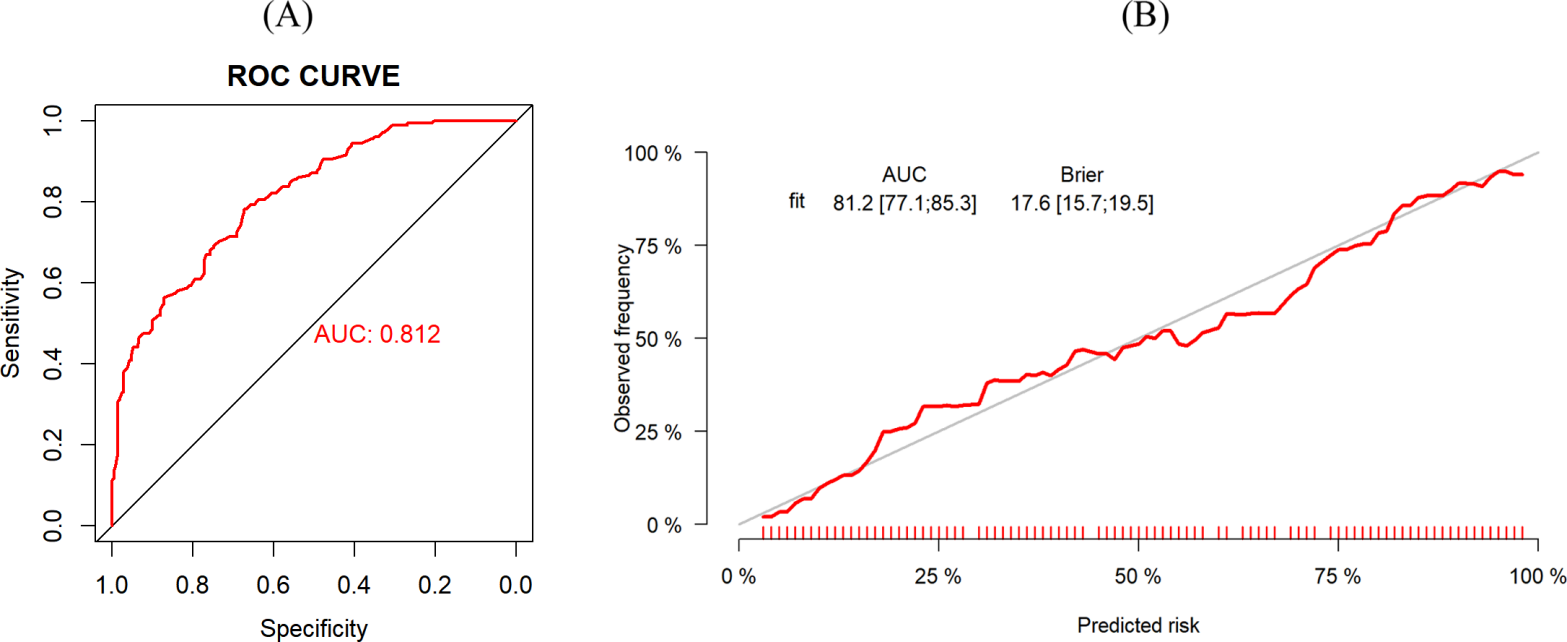
**(A)** ROC curve for risk prediction model for training group. **(B)** Calibration curves of the training group.

**Figure 4:**
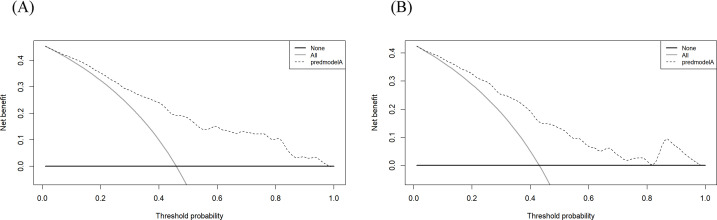
**(A)** Decision curve analysis of the training group. **(B)** Decision curve analysis of the validation group.

The predictive performance of a model is evaluated from three aspects: discrimination, calibration, and clinical utility. The Bootstrap method was employed for repeated sampling (1,000 iterations) to internally validate the training group data. The optimal cutoff value of the model was determined by maximizing the Youden index. The optimal cutoff value for this model was 0.409, at which the sensitivity and specificity were 0.782 and 0.673, respectively. The AUC was 0.812 (95% CI: 0.771–0.853) (**Figure 3A**). The Brier score was 0.176 (95% CI: 0.157–0.195). The results of the calibration curve suggested that the probability of oral frailty predicted by the model was in high agreement with the actual probability of oral frailty (**Figure 3B**), and the prediction was more accurate. Hosmer-Lemeshow test χ^2^ value = 12.382, *P* = 0.193, indicating that there was no statistically significant difference between the predicted risk and the actual risk. In addition, the results of the decision curves (**Figure 4A**) showed that the use of the model to make clinical decisions resulted in a net greater benefit than “no intervention” or “all intervention” options across probability thresholds of 0.01 to 0.99.

**Figure 5:**
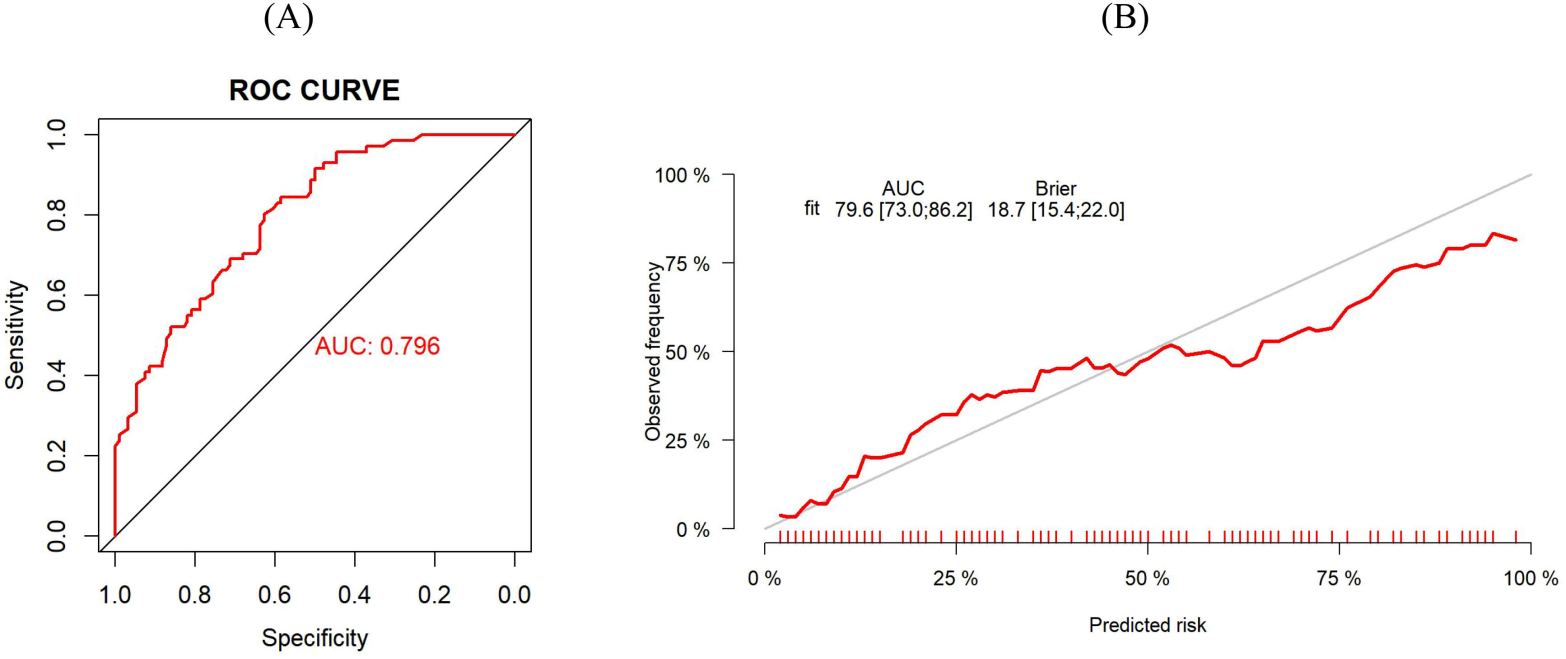
**(A)** ROC curve for risk prediction model for validation group. **(B)** Calibration curves of the validation group.

The AUC value of the validation group was 0.796 (95% CI: 0.730–0.862) (**Figure 5A**), indicating that the model still had good discrimination in the external validation. The Brier score was 0.187 (95% CI: 0.154–0.220), and the calibration curve results showed that the predicted probability of oral frailty in the model was in good agreement with the actual probability of oral frailty in the validation group (**Figure 5B**). The Hosmer-Lemeshow test results were: χ^2^ value = 14.922, *P* = 0.093, which represented that the model had a good degree of fitting between the expected probability and the actual probability in the validation group. The results of the decision curves are shown in **Figure 4B**, and the use of this model provides a net benefit to clinical practice at threshold probabilities ranging from 0.01 to 0.99.

### 3.4 Drawing of the nomogram

**Figure 6:**
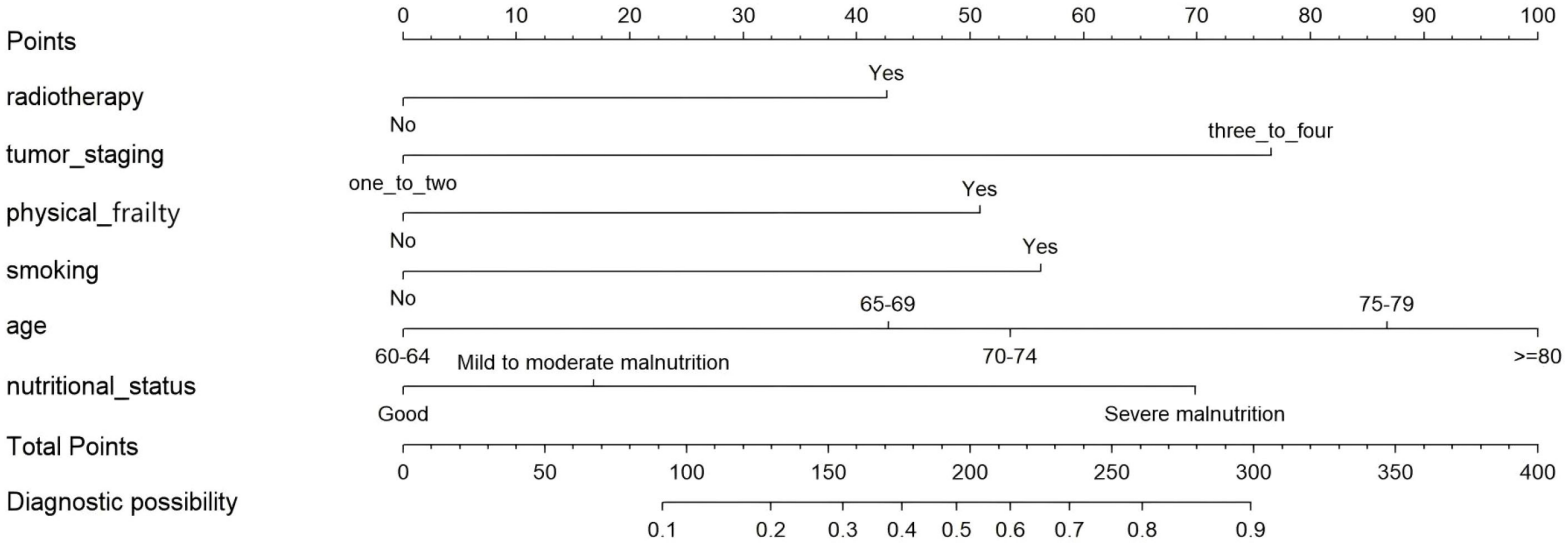
Nomogram for risk prediction of oral frailty.

In this study, the R software 4.4.3 “rms” package was used to construct a nomogram model for predicting the occurrence of oral frailty in elderly patients with EC, as shown in **Figure 6**. Application methods of the nomogram model: According to the variables of oral frailty in elderly patients with EC, the corresponding line segment was selected to make a vertical line downward to obtain the corresponding score value of each variable. According to this principle, the risk scores of all predictive factors were calculated in turn, and the corresponding prediction probability was found after the scores were added up, so as to obtain the risk probability of oral frailty in elderly patients with EC.

**Figure 7:**
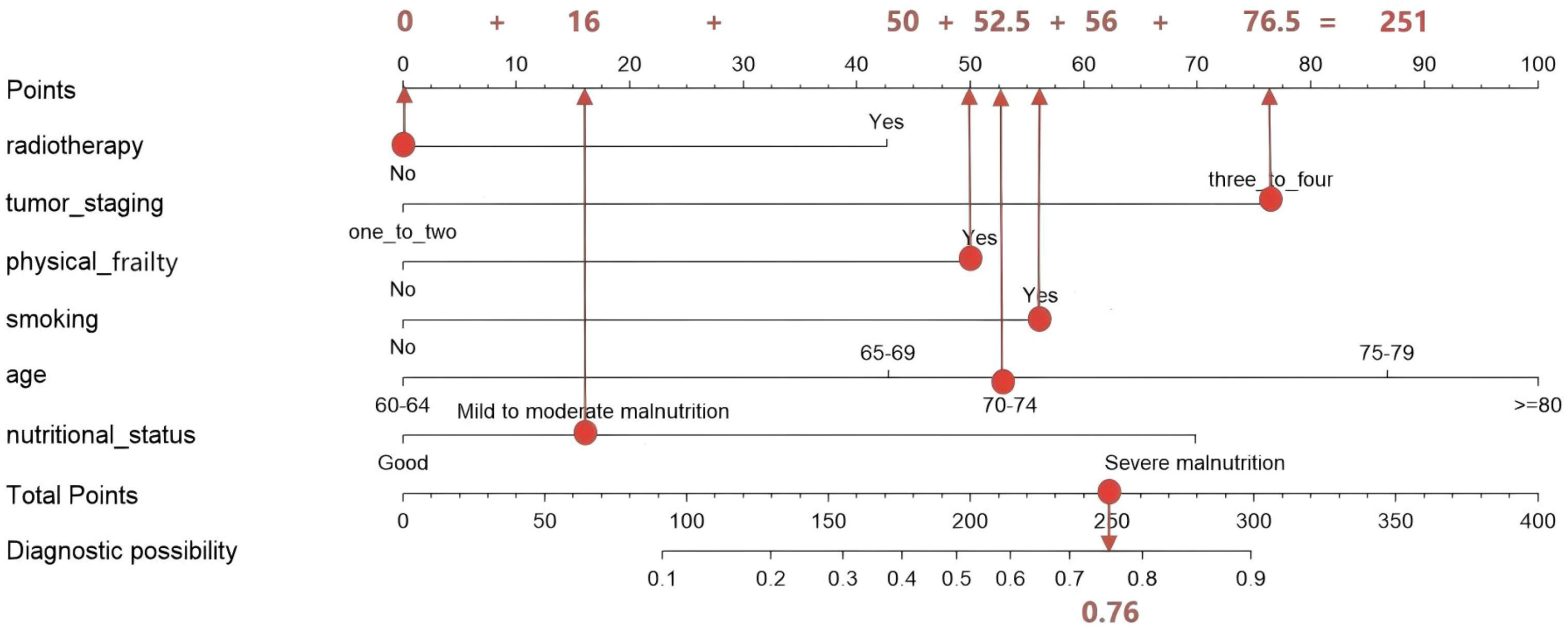
Example of using the oral frailty risk prediction nomogram.

Nomogram application example: A 71-year-old patient with EC has no radiotherapy history, tumor staging IIIt presents with physical frailty, a smoking history, moderate malnutrition, and is 71 years old. Based on the above characteristics, this nomogram was used to predict the probability of oral frailty occurrence. The specific operation is shown in **Figure 7**: ① Radiotherapy status dimension: Locate the “No” label on the corresponding axis, draw a vertical line upward from this position to intersect with the top Points axis, and the intersection point corresponds to a score of 0; ② Tumor staging dimension: Position the “three-or-four” option on this axis, draw a vertical line upward to intersect with the Points axis, and the intersection point corresponds to a score of 76.5; ③ Physical frailty dimension: Locate the feature “presents with physical frailty” and draw a vertical line upward, which intersects with the Points axis at a score of 50; ④ Smoking status dimension: Since the patient has a smoking history, draw a vertical line according to the corresponding label to intersect with the Points axis, and the intersection point corresponds to a score of 56; ⑤ Age dimension: Locate 71 years old on the corresponding axis, draw a vertical line upward to intersect with the Points axis, and the intersection point corresponds to a score of 52.5; ⑥ Nutritional status dimension: Locate the “Mild to moderate malnutrition” label on the axis, draw a vertical line upward to intersect with the Points axis, and the intersection point corresponds to a score of 16. Sum the scores from the six predictors to obtain a total score of 251. Locate the value 251 on the Total Points axis of the nomogram, draw a vertical line downward from this position to intersect with the bottom Predict Value axis, and the intersection point corresponds to a value of approximately 0.76. Thus, the predicted probability of oral frailty for this patient is approximately 76%.

## 4 Discussion

A total of 390 elderly patients with EC were included in the training group of this study, and 179 cases had oral frailty, with an incidence rate of 45.90%. A total of 165 elderly patients with EC were included in the validation group, and 71 cases had oral frailty, with an incidence of 43.03%. There were 555 elderly patients with EC and 250 patients with oral frailty in the two groups, with an incidence rate of 45.05%. The prevalence of oral frailty in the present study was higher than that reported in Yamamoto’s study (28), The reasons for the differences are as follows: the study population is elderly patients with EC. On the one hand, tumor invasion destroys the normal anatomical structure of the esophagus, causing luminal stenosis or even obstruction, stimulating the nerve endings of the esophageal wall, causing dysphagia, pain, oral mucositis and other symptoms, and increasing the risk of oral infection. On the other hand, cancer-related treatments, such as radiotherapy, chemotherapy, and targeted therapy, can reduce the body’s immunity, cause oral bacterial translocation and colonization, cause oral microbial flora imbalance, and cause inflammatory or ulcerative changes in the oral mucosa (29), finally, it leads to the occurrence of oral frailty. According to the survey of this study, the incidence of oral frailty in elderly patients with EC is high. It is recommended that medical and nursing staff carry out routine oral frailty screening for elderly patients with EC, provide oral health knowledge related to comprehensive and multiple measures, identify patients with high risk of oral frailty in this population as soon as possible, and strengthen the management of high-risk patients and risk factors of oral frailty. For patients with oral frailty, oral health care and medical services should be actively provided to meet the oral health and health needs of elderly patients with EC, and timely intervention should be performed to reduce the occurrence of adverse health outcomes.

This study found that radiotherapy history is an independent risk factor for the development of oral frailty in elderly patients with EC, which is consistent with previous studies (30, 31). Its mechanism is closely associated with the specific characteristics of radiotherapy for EC. Due to the anatomical proximity, the radiation fields for EC are highly likely to cover major salivary glands such as the parotid glands and submandibular glands, resulting in irreversible damage to their acinar cells, accelerating the apoptosis of oral mucosal cells, and impairing their repair capacity, thereby triggering oral mucositis (32, 33). In addition, severe oral mucositis caused by the direct radiation-induced damage to the oral mucosa is often accompanied by bleeding, pain, and oral infections, leading to dysphagia and communication disorders, which accelerates the progression of oral frailty. Therefore, medical staff should pay greater attention to patients undergoing radiotherapy, conduct regular examinations of oral mucosal status, and provide preventive education and individualized interventions. Meanwhile, patients should be encouraged to perform oral function exercises, maintain oral hygiene, and adopt other relevant behaviors to prevent or delay the occurrence and progression of oral frailty.

Tumor staging is commonly used to evaluate the degree of malignancy of EC in elderly patients. The results of this study showed that the risk of oral frailty in elderly EC patients with tumor staging III–IV was 5.059 times that of patients with tumor staging I–II, that is, the higher the tumor staging, the greater the possibility of oral frailty. The results were similar to those of previous studies (34). The reason may be that tumor staging reflects tumor burden, invasion and systemic influence. Patients with staging III–IV are more active in tumor biological behavior, with high malignant degree and obvious invasion of tumor cells. Tumor cells break through the submucosa and infiltrate into the muscular layer and even the outer membrane of the esophagus, resulting in increased stenosis of esophageal lumen and aggravation of dysphagia. Eating restriction directly affects the nutritional intake of patients, which in turn has a negative impact on the normal metabolism and functional maintenance of oral tissues. On the other hand, patients with staging III-IV tumors are mostly advanced patients, with increased secretion of inflammatory factors (such as IL-6, TNF-α, etc.), and various cytokines and inflammatory mediators released will trigger systemic inflammatory response (35), it can damage the local oral microenvironment and affect oral tissue metabolism. The results of this study remind clinicians to pay attention to tumor staging as a potential risk factor. For patients with middle and advanced EC, while carrying out effective symptom management, elderly patients with EC should be guided to carry out daily oral training or provide them with oral health management services, so as to slow down the process of oral frailty.

Among 390 elderly patients with EC included in the training group of this study, 249 patients had physical frailty, and 141 of them had oral frailty, with an incidence of 56.63%. Univariate and multivariate analysis showed that elderly EC patients with physical frailty had a higher risk of oral frailty, which was highly consistent with the results of previous studies (36, 37). The analysis may be due to the limitation of esophageal function in elderly patients with EC, which affects the intake and absorption of nutrients, dysphagia and negative nitrogen balance caused by tumor consumption, leading to systemic weakness, reducing the body’s immunity and the repair ability of oral tissue. Oral mucosal injury is difficult to heal, and oral function gradually decreases, thus promoting the occurrence and development of oral frailty (37). On the other hand, physical frailty can affect the self-care ability of patients. Due to the decline of physical function, elderly patients are more difficult to carry out effective oral cleaning and nursing in the state of late-stage systemic frailty, which leads to the deterioration of oral hygiene, the breeding of oral bacteria, further damage to oral function, and accelerate the process of oral frailty. In addition, existing studies have shown that (38), decreased physiological reserve, reduced activity and physical strength, reduced social range with the elderly, reduced opportunities for oral communication, and reduced movement of oral and maxillofacial muscles and tongue muscles may also contribute to oral frailty. Nursing staff can prevent or delay the development of oral frailty through effective comprehensive management of frailty, such as exercise, nutrition management, psychological intervention, and so on. Nurses can also encourage patients to actively participate in social activities, increase the movement of oral and maxillofacial muscles and tongue, and delay the development of oral frailty.

The results of this study indicate that smoking history is an independent predictor of oral frailty in elderly patients with EC, which is similar to the findings of previous studies (36, 39). The reason may be that nicotine in tobacco can significantly promote the activity of Streptococcus mutans and accelerate the formation of dental caries (40, 41). In addition, long-term smoking can cause adverse stimulation to the oral environment, which can easily lead to gingival bleeding, reduced dental stability and oral ulcers (42), causes oral frailty. The harm of smoking has been confirmed in a variety of diseases, and the harm of smoking to health has been generally recognized by the public. However, 42.70% of elderly patients with EC in this study still had a smoking history. For this reason, healthcare providers should incorporate smoking cessation intervention programs into the comprehensive disease management of EC. Efforts should be made to enhance these patients’ understanding of smoking cessation through multiple channels such as health lectures and online communities, conduct in-depth smoking cessation publicity and education, and provide them with diversified, comprehensive, and targeted effective smoking cessation support. This aims to improve the success rate of smoking cessation among patients, thereby optimizing oral health and reducing the incidence of oral frailty.

This study found that advanced age is an independent predictor of oral frailty in elderly patients with EC, which is basically consistent with many studies at home and abroad (43–45). The reasons may be as follows: first, with the increase of human tissue structure and physiological function, the amount of saliva secretion in the elderly gradually decreases, coupled with the loss of calcium in bones, gingival atrophy, tooth root exposure, and dental nerve degeneration occur gradually, leading to the decline of oral self-purification ability and oral resistance, thus increasing the risk of oral frailty. Second, the awareness of oral health care in elderly patients is poor, and most of them are affected by traditional concepts. When oral problems occur, they often deal with them or even do not deal with them, and they do not need to go to the hospital for examination and treatment. Lack of oral health knowledge, such as periodontal disease and its risk factors, the time of toothbrush replacement, and the use of dental floss, leads to delay in seeking medical treatment (46); thirdly, the self-care ability of daily life of elderly patients with EC is generally poor, the movement is slow, and the oral cleaning is not accurate and timely, which leads to the accumulation of oral bacteria and the formation of dental plaque, leading to poor oral health. This may also be another reason why the elderly are more susceptible to oral frailty. Therefore, medical and nursing staff should focus on elderly patients with EC, carry out multi-directional and multi-level oral frailty screening for elderly patients with EC at different ages, and guide patients to improve their oral health status through diet adjustment and effective oral functional exercise according to their age, disease condition and oral condition.

The results of this study showed that among 179 elderly EC patients with oral frailty in the training group, 71 patients (39.66%) had severe malnutrition. Further analysis showed that the risk of oral frailty in elderly EC patients with severe malnutrition was 4.391 times higher than that in patients with good nutrition (OR = 4.391, 95% CI: 2.276–8.471), and severe malnutrition was an independent risk factor for oral frailty in patients (*P*< 0.001). This is consistent with the results of several studies (47, 48). Esophageal dysfunction directly affects the eating status of patients, leading to the limitation of the type and quantity of food intake, which leads to the insufficient intake of proteins, vitamins, minerals and other nutrients (49). Long-term nutritional deficiency can cause negative nitrogen balance in the body, accelerate the breakdown of muscle protein, lead to the reduction of muscle mass and muscle strength, aggravate the general frailty, and impair the repair ability of oral tissue and oral function at multiple levels (50). On the one hand, the lack of nutrition makes oral mucosal cells unable to obtain sufficient nutritional support, and the rate of cell renewal and repair slows down. On the other hand, the body’s immune function is reduced in the frailty state, and the resistance to oral infection is weakened, which further aggravates oral lesions. In addition, nutritional status can also affect the ability of elderly patients with EC to take oral care, leading to the deterioration of oral hygiene, forming a vicious circle of oral dysfunction and malnutrition promoting each other. Therefore, medical and nursing staff should pay attention to and timely assess the nutritional status of elderly patients with EC. Elderly patients with malnutrition or risk of malnutrition should receive dietary fortification between meals, and elderly patients with swallowing and chewing difficulties can choose fortified foods with improved texture to ensure adequate energy intake, and parenteral and parenteral nutrition if necessary.

In this study, the validation method was chosen to construct and internally validate the OF risk prediction model in the elderly patients with EC in the First Affiliated Hospital of Naval Medical University, and to externally validate the model in the elderly patients with EC in Qingdao Hiser Hospital Affiliated of Qingdao University to ensure the stability and extrapolation of the model. The predictive efficacy of the model was evaluated from three aspects: discrimination, calibration and clinical validity. The AUC value of internal validation was 0.812 (95% CI: 0.771–0.853), and the AUC value of external validation was 0.796 (95% CI: 0.730–0.862). Although it was lower than the internal validation, it still reached the performance standard of the model. The internal and external validation results showed that the model had a strong ability to distinguish whether elderly patients with EC had oral frailty. The prediction model constructed in this study had good calibration. The Hosmer-Lemeshow goodness of fit test, calibration curve and Brier score were used to comprehensively evaluate the calibration degree, and the results of the training group and the validation group showed that the prediction accuracy of the model was good. In addition, the net benefit curve corresponding to the risk prediction model of this study was consistently higher than the strategy of not intervening in any patients within the range of threshold probability from 0.01 to 0.99. This indicates that within this threshold probability range, using this model to guide clinical interventions is more likely to yield practical net benefits for patients compared with the strategy of not intervening in any patients, and thus is of clinical decision-making value. In conclusion, the performance of the prediction model constructed in this study meets the standard in the internal and external validation, and it can be used to accurately predict the risk of oral frailty in elderly patients with EC. At the same time, the six predictors in the model constructed in this study are all routine clinical monitoring indicators, and the project data are easy to measure and obtain, which further improves the clinical accessibility of the model and provides a guarantee for the smooth development of the clinical application of the prediction model, with strong practicability.

The model developed in this study can effectively predict the risk of oral frailty in elderly patients with EC, which is simple, cost-effective and practical, and provides guidance for clinical practice. Nomogram is a graphical expression of a model, which can be used to analyze the risk of patients intuitively and conveniently. It is one of the important presentation methods of clinical prediction models. In this study, R4.4.3 software was used to establish a nomogram model to predict the occurrence of oral frailty in elderly patients with EC. The model was visualized and displayed, which could visually display the hazard ratios of predictors in the logistic regression model, allow direct calculation of the probability of target events, and help for early screening of elderly patients with EC. To facilitate clinical application, this study clearly illustrates how to quickly assess patients’ risk scores using a nomogram through examples and visualizations (**Figure 7**), enabling clinicians to directly apply it in practice. The model constructed in this study helps medical staff early identify and predict high-risk groups for oral frailty, and promptly implement preventive and nursing measures, thereby achieving early prevention and intervention and reducing the incidence of oral frailty.

## 5 Limitations

The limitations of this study are as follows: first, the sample size of this study is limited, and only elderly patients with EC from one Class III Grade A hospital in Shanghai and one Class III Grade A hospital in Qingdao were selected for investigation. The results may have selection bias, so the conclusions need to be further verified when extrapolated to other places and regions. Second, although this study used time-series data from the training group and validation group, it is essentially a cross-sectional study design, which can only provide evidence of risk associations between potential factors and oral frailty, but cannot directly evaluate the model’s predictive ability for long-term outcomes and intervention effects. Future studies could benefit from a prospective longitudinal study design to better understand the dynamic interactions between these variables over time. Finally, the selection of candidate predictor variables for the model is limited. In this study, only radiotherapy history and chemotherapy history were included as dichotomous variables in the analysis, while specific objective indicators such as total radiation dose, fractionation regimen, irradiation field, as well as types and cycles of chemotherapy drugs failed to be collected and analyzed. Incorporating these objective indicators may further improve the model’s performance. Future research should include more objectively measured predictors to more comprehensively and objectively identify the risk factors for oral frailty. In conclusion, multi-center and large-sample studies are needed to improve the comprehensive collection of variables and sampling representativeness in the future. At the same time, multi-model modeling comparison such as machine learning can be considered to screen the optimal model and provide more accurate prediction tools. In addition, the development of dynamic nomograms or online calculators may also be considered to establish a prediction platform to promote the screening, prevention, and management of oral frailty in elderly patients with EC.

## 6 Conclusions

In this study, LASSO regression and multivariate analysis were used to screen the risk factors most related to oral frailty in elderly patients with EC. The key predictors included radiotherapy history, tumor staging III–IV, physical frailty, smoking history, advanced age, and poor nutritional status. The risk prediction model was constructed to quantitatively assess the risk of oral frailty in this population. Internal and external validation showed that it had good discrimination, calibration and clinical validity, and the model was presented in the form of a nomogram. The prediction model provides important guidance for clinical work, provides a visual quantitative risk assessment tool for medical staff to evaluate oral frailty, and provides strong data support for the precise screening of high-risk groups.

## Data Availability

The raw data supporting the conclusions of this article will be made available by the authors, without undue reservation.
